# Variation in heart rate influences the assessment of transient ischemic dilation in myocardial perfusion scintigraphy

**DOI:** 10.1186/1471-2385-7-1

**Published:** 2007-01-11

**Authors:** William D Leslie, Daniel P Levin, Sandor J Demeter

**Affiliations:** 1 Department of Medicine, University of Manitoba, Winnipeg, Canada; 2 Section of Nuclear Medicine, University of Manitoba, Winnipeg, Canada

## Abstract

**Background:**

Transient arrhythmias can affect transient ischemic dilation (TID) ratios. This study was initiated to evaluate the frequency and effect of normal heart rate change on TID measures in routine clinical practice.

**Methods:**

Consecutive patients undergoing stress/rest sestamibi gated myocardial perfusion scintigraphy were studied (N = 407). Heart rate at the time of stress and rest imaging were recorded. TID ratios were analyzed in relation to absolute change in heart rate (stress minus rest) for subjects with normal perfusion and systolic function (Group 1, N = 169) and those with abnormalities in perfusion and/or function (Group 2, N = 238).

**Results:**

In Group 1, mean TID ratio was inversely correlated with the change in heart rate (r = -0.47, P < 0.0001). For every increase of 10 BPM in heart rate change, the TID ratio decreased by approximately 0.06 (95% confidence interval 0.04–0.07). In Group 2, multiple linear regression demonstrated that the change in heart rate (beta = -0.25, P < 0.0001) and the summed difference score (beta = 0.36, P < 0.0001) were independent predictors of the TID ratio.

**Conclusion:**

Normal variation in heart rate between the stress and rest components of myocardial perfusion scans is common and can influence TID ratios in patients with normal and abnormal cardiac scans.

## Background

The assessment of myocardial perfusion with radioactive flow tracers provides valuable clinical information for diagnosis and risk stratification [1-3]. Individuals with normal myocardial perfusion during an adequate stress procedure are generally regarded as being at low risk for major cardiac events [4,5]. In addition to perfusion, a number of ancillary functional parameters can be extracted from these images [6]. These have been shown to provide additional prognostic information that is additive to the clinical and perfusion data alone. For example, left ventricular systolic function can be assessed with gated single photon emission computed tomography (SPECT), left ventricular cavity size can be measured, and lung uptake measurements can be computed [7-11]. Each of these parameters has been shown to provide incremental prognostic information.

"Transient ischemic dilation" (TID) of the left ventricular cavity is a marker of severe coronary artery disease and adverse outcomes [12,13]. Elevated TID measures have also been correlated with cardiac events in individuals with normal perfusion patterns [14]. Since average left ventricular cavity volume is influenced by heart rate (faster heart rate leading to smaller volumes due to reduced filling time) it is possible that TID measurements may be affected by normal change in heart rate between the stress and rest images. Falsely elevated and reduced TID ratios have been reported in individuals experiencing transient arrhythmias [15]. This study was initiated to evaluate the frequency and effect of normal heart rate change on TID measures in routine clinical practice.

## Methods

### Patient population

Consecutive patients referred to the Nuclear Medicine Department at St. Boniface General Hospital for gated SPECT stress/rest sestamibi myocardial perfusion scintigraphy between February 2005 and July 2005 were studied. We excluded individuals who did not complete both stress and rest imaging (n = 1), who had persistent or intermittent arrhythmia (n = 6), or where there was missing heart rate information at the time of the scan acquisition (n = 41). Patient consent was not required since all procedures were performed as part of routine clinical imaging. This manuscript was reviewed and approved in accordance with the facility's Office of Clinical Research.

### Image acquisition and processing

The procedures used in our Nuclear Medicine laboratory have been previously reported [16]. A two-day protocol (gated rest with non-gated stress) utilizing treadmill exercise is the preferred procedure in our laboratory. Whenever possible, beta blockers and calcium-channel antagonists are withheld for 24–48 hours prior to the stress procedure, and nitrates are avoided for at least six hours. Symptom-limited treadmill exercise is performed with ^99m^Tc-sestamibi injection (500–600 MBq) at peak exercise followed by 1–3 minutes of exercise post-injection. For individuals unable to achieve a satisfactory exercise workload, dipyridamole pharmacologic stress is used (0.56 mg/kg administered intravenously over 4 minutes, with tracer injection 4 1/2 minutes later). Low-level supplementary exercise is performed whenever possible following dipyridamole infusion for those patients without a left bundle branch block. Supine, gated SPECT imaging is commenced 30–60 minutes post-stress and 45–75 minutes following the resting tracer injections (ADAC Forte, Phillips, Milpitas, CA). A one-day procedure (non-gated rest followed by gated stress) is used in a small number of cases. In these patients the rest imaging with ^99m^Tc-sestamibi (250–300 MBq) was performed in the morning followed by stress imaging (1000 MBq) 2–3 hours later.

Heart rate was recorded immediately prior to stress and rest image acquisition. When the patient was attached to an EKG monitor for purposes of gating, the heart rate displayed by the monitor was recorded. For the non-gated scan (usually stress), the technologist counted the radial pulse for 15 seconds and then multiplied this by 4 to determine heart rate. Change in heart rate was calculated as heart rate at the time of stress imaging minus heart rate at the time of rest imaging. This was subsequently grouped into tertiles.

Image data was processed using commercially available software (AutoSPECT and QPS/QGS AutoQUANT, Cedar Sinai Medical Centre and ADAC Laboratories, Milpitas, CA) [17-19]. Left ventricular contours were checked visually and manually adjusted if the computer-generated automatic contours were found to be incorrect. Briefly, this software provides a quantitative defect extent and severity measurement, the summed stress score (SSS), defined from gender-specific normal limits by adding the scores from twenty left ventricular segments (0 = normal to 4 = absent uptake) on the stress sestamibi images. [20,21]. The summed rest scores (SRS) and summed difference scores (SDS) provide indices of infarction and ischemia, respectively. We have previously shown that there is close agreement between the visual and automated quantitative assessment in terms of diagnosis and prognosis [22,23].

An automatic calculation of left ventricular volumes post-stress and at rest was obtained using a validated algorithm that operates in three-dimensional space on the reconstructed short-axis image sets [24]. The TID ratio is derived from the endocardial volumes as the ratio of the average left ventricular volume at stress divided by the average volume at rest. Left ventricular ejection fraction (LVEF) was derived from 8-frame gated SPECT using the same validated commercial software package [25].

### Image interpretation

Interpretation of the scan data was performed by nuclear medicine specialists with extensive experience in cardiac nuclear medicine. Scans were jointly reported by two physicians, and if necessary a third physician was involved to reach a consensus. Images were categorized as *normal*, *equivocal*, *abnormal with fixed defects*, *abnormal with fully reversible defects*, or *abnormal with partially reversible defects*. For analytic purposes, these categories were recoded using two variables as follows: normal scan (normal or equivocal) versus abnormal scan (abnormal with fixed or reversible defects); no reversibility (normal, equivocal or abnormal with fixed defects) versus reversibility (abnormal with fully reversible or partially reversible defects).

The patient population was divided into two groups, one with normal perfusion and function (Group 1) and the other with abnormal perfusion and/or function (Group 2). For classification purposes, normal was defined as a visual perfusion interpretation of normal or equivocal, SSS ≤ 3 and LVEF ≥ 45% [26]. All criteria had to be satisfied. This group was used to assess the effect of heart rate change on TID ratio in individuals without detectable ischemia or impaired systolic function. Group 2 was used to assess the effect of heart rate change on TID ratio categorization in patients with abnormal perfusion and/or systolic function. Nominal upper limits of normal TID ratios (mean + 1.96 standard deviations) were derived from Group 1 overall and by tertile in absolute change in heart rate. TID ratios in Group 2 were categorized according to these cutoffs to determine the effect of using a heart rate stratified normal range.

### Statistical analysis

The absolute change in heart rate (stress heart rate minus rest heart rate) was studied in relation to the automated TID ratio. The correlation between heart rate change and TID ratio was assessed in the two study groups separately using Pearson correlation coefficients. Multivariable linear regression was used to assess the independence of the relationship between TID ratio, heart rate change and ischemic burden as measured by the SDS. Heart rate change and TID ratio were normally distributed, and graphical analysis of the residuals was consistent with a linear relationship. Continuous variables are reported as mean ± SD, and P < 0.05 is considered to represent a statistically significant difference. Group comparisons were performed using ANOVA (continuous variables) or a Chi-square test (categorical variables). Post hoc pairwise comparisons were performed with Fisher's least significant difference procedure. Statistical analysis was performed with a commercial software package (Statistica Version 6.1, StatSoft Inc, Tulsa, OK).

## Results

### Overall population

The study cohort consisted of 407 patients, of whom 169 (42%) had normal perfusion and systolic function (Group 1) with the remaining 238 (58%) having abnormal perfusion and/or systolic function (Group 2). Patient characteristics are summarized (Table 1). As expected, there were significant differences between these groups. Differences in variables related to perfusion and systolic function were in keeping with the group definitions. Group 1 patients tended to be younger (P = 0.06), more likely to be female (P < 0.0001), and less likely to require pharmacologic stress (P < 0.0001). The majority (86%) of the scans were performed as two-day procedures with gating of the rest scan. The overall mean change in heart rate (stress – rest) was 6 ± 9 BPM. Heart rate change was slightly greater in Group 1 than Group 2 (8 ± 10 vs. 5 ± 9, P = 0.005). A lower mean TID ratio was seen in Group 1 than Group 2 (1.01 ± 0.12 vs. 1.06 ± 0.12; P = 0.0001).

**Table 1:** Patient demographics and characteristics. Group 1 had normal perfusion and function. Group 2 had abnormal perfusion and/or function.

	All Patients N = 407	Group 1 N = 169	Group 2 N = 238	P value *
Age (years)	65 ± 12	63 ± 13	66 ± 11	0.06
Male (percent)	213 (52%)	69 (41%)	144 (60%)	<0.0001
Height (cm)	168 ± 10	166 ± 9	170 ± 10	0.005
Weight (kg)	83.6 ± 16.9	81.4 ± 16.7	85.2 ± 17.0	0.03
Scan protocol (percent)				
Two-Day	351 (84%)	148 (88%)	202 (85%)	>0.2
Same-Day	56 (14%)	21 (12%)	35 (15%)	
Stress method (percent)				
Exercise only	173 (43%)	100 (59%)	73 (31%)	<0.0001
Dipyridamole	234 (57%)	69 (41%)	165 (69%)	
Heart rate (BPM)				
Stress scan	70 ± 12	71 ± 13	70 ± 13	0.15
Rest scan	64 ± 12	64 ± 11	64 ± 13	>.2
Change	6 ± 9	8 ± 10	5 ± 9	0.005
Perfusion				
SSS	7 ± 8	1 ± 1	11 ± 8	<0.0001
SRS	3 ± 6	0 ± 0	5 ± 7	<0.0001
SDS	4 ± 4	1 ± 1	6 ± 5	<0.0001
LVEF (%)	57 ± 14	64 ± 9	52 ± 15	<0.0001
TID Ratio	1.04 ± 0.12	1.01 ± 0.12	1.06 ± 0.12	0.0001

* Group 1 vs. Group 2

In the combined study population (Groups 1 and 2) we looked for factors associated with the difference in heart rate between stress and rest. Larger changes were inversely associated with age (r = -0.19, P < 0.001) and smaller left ventricular cavity volume on the stress image (r = -0.11, P = 0.026). Individuals undergoing pharmacologic stress showed a smaller difference in heart rate than those undergoing exercise (mean change 4 ± 8 BPM versus 10 ± 10 BPM, P < 0.0001). Scan protocol also affected change in heart rate, with a smaller change in those undergoing same-day scanning (mean change 2 ± 8 BPM) than when a two-day protocol was used (9 ± 9 BPM; P = 0.0004). Visually abnormal perfusion, visual reversibility, SSS, SDS, and LVEF were unrelated to change in heart rate.

Since determinants of TID ratio were anticipated to be quite different for subjects with normal cardiac perfusion and function (Group 1) and those with cardiac abnormalities (Group 2), these two groups were analyzed separately.

### Normal cardiac perfusion and function (Group 1)

**Figure 1:**
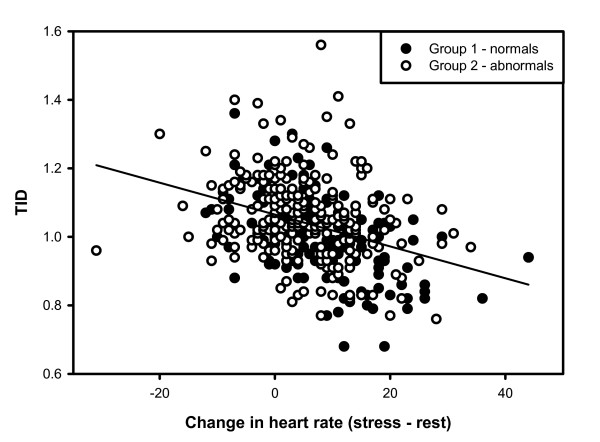
Transient ischemic dilation (TID) ratio in relation to change in heart between stress and rest imaging for Group 1 (normal cardiac perfusion and function) and Group 2 (abnormal cardiac perfusion and/or function).

There was a significant negative correlation between heart rate change and the TID ratio in Group 1 (r = -0.47, p < 0.0001). For every increase of 10 BPM in heart rate change, the TID ratio decreased by approximately 0.06 (95% confidence interval 0.04–0.07) (Figure 1). This association was demonstrated for both exercise and pharmacologic stress (r = -0.56, P = 0.0007 and r = -0.41, P = 0.001 respectively), with mean TID ratios that were significantly different (0.98 ± 0.11 and 1.05 ± 0.12 respectively, P = 0.0003). Significant correlations between heart rate change and the TID ratio were also seen for patients undergoing one-day (r = -0.53, P = 0.013) or two-day (r = -0.45, P < 0.0001) protocols.

**Figure 2:**
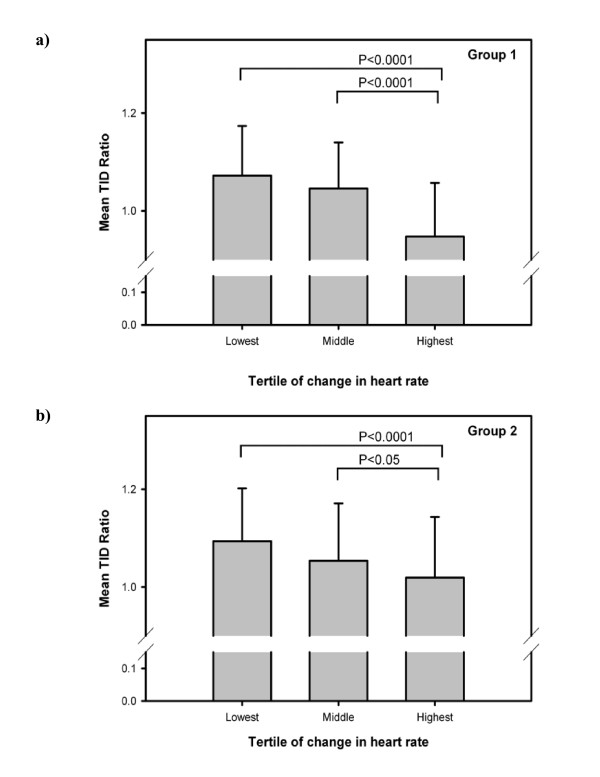
**Effect of heart rate change on mean transient ischemic dilation (TID) ratio**. (a) Group1 consists of patients with normal cardiac perfusion and function. (b) Group 2 consists of patients with abnormal cardiac perfusion and/or function.

When heart rate change was grouped into tertiles (stress minus rest lowest tertile less than -2 BPM, highest tertile greater than 8 BPM), a strong effect on mean TID ratio was demonstrated (ANOVA P < 0.0001). Post hoc comparisons (Figure 2) showed that individuals in the highest tertile of change in heart rate differed significantly from the other two groups (P < 0.0001).

### Abnormal cardiac perfusion and/or function (Group 2)

Of the 238 cases, an inverse correlation was again noted between change in heart rate and TID ratio (r = -0.25, P < 0.0001). Mean TID ratios were significantly different between patients undergoing exercise (1.03 ± 0.13) or pharmacologic stress (1.07 ± 0.11 respectively, P = 0.023), and between patients undergoing one-day (1.11 ± 0.09) or two-day protocols (1.05 ± 0.12 respectively, P = 0.0021). Other factors that correlated with TID ratio were SSS (r = 0.25, P < 0.0001) and SDS (r = 0.36, P < 0.0001). Multiple linear regression confirmed the independent effects of change in heart rate (beta = -0.25, P < 0.0001) and SDS (beta = 0.36, P < 0.0001). Significant differences in the mean TID ratio were seen when heart rate change was grouped into tertiles as previously defined (ANOVA P < 0.0001). Individuals in the highest tertile of change in heart rate differed significantly from the other two groups (P < 0.05).

### Heart rate stratified categorization

The upper limit of normal TID ratio derived by pooling all patients in Group 1 was 1.24. Upper limits of normal for each of the previously defined tertiles of change in heart rate were 1.27 for the lowest tertile, 1.23 for the middle tertile, and 1.16 for the highest tertile. The TID ratio for Group 2 exceeded either the pooled or the rate stratified cutoff in 21 cases. Of these, 8 (38%) showed discordance between the two approaches (6 elevated with the rate stratified range but normal using the pooled cutoff, and 2 normal with the rate stratified range but elevated with a pooled cutoff).

It is also possible to derive a TID correction for heart rate change based upon the linear regression for Group 1 (TID = 1.054 - .0058 * [heart rate change]) which effectively corrects the TID ratio to a heart rate change of 0. The upper limit of normal TID ratio decreased from 1.24 (uncorrected) to 1.21 (corrected), the more narrowly defined normal range in keeping with removal of a source of variability. Seven Group 2 cases showed elevated TID ratios with the rate adjusted reference range but normal ratios using the uncorrected range.

**Figure 3:**
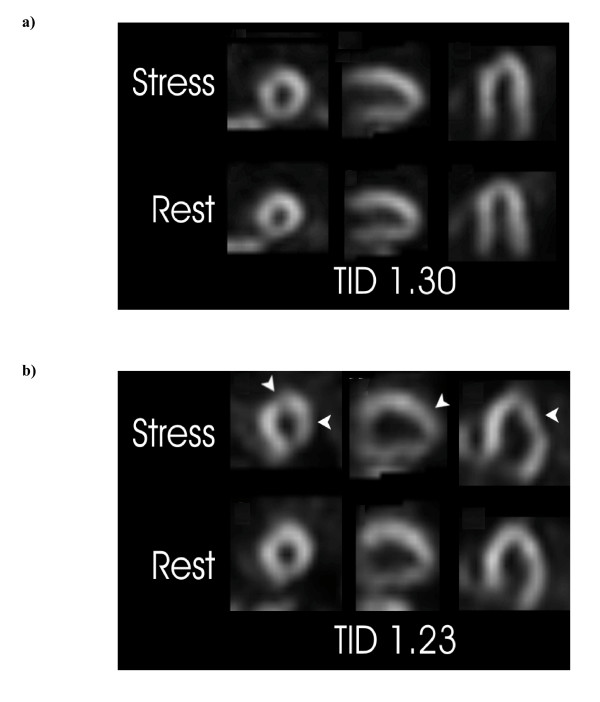
**Illustrative cases demonstrating how change in heart rate can affect transient ischemic dilation (TID) ratios**. (a) Low-risk left ventricular perfusion pattern (equivocal in the posterobasal segment due to possible attenuation artifact) with an elevated TID ratio. Heart rate was slower with stress than with rest imaging (78 BPM and 98 BPM, respectively, with absolute difference -20 BPM). (b) Severely abnormal left ventricular perfusion with a borderline TID ratio. Multiple reversible defects (arrowheads) indicate multivessel ischemia. Heart rate was faster with stress than with rest imaging (75 BPM and 60 BPM, respectively, absolute difference 15 BPM) and the TID ratio was abnormal using a rate stratified normal range.

Two examples are presented that illustrate opposite effects of change in heart rate (Figure 3). In one case, heart rate change (slower with stress) was felt to produce an artifactually elevated TID ratio. The clinical information and perfusion pattern in this case did not support severe ischemia. In the other case, heart rate change (faster with stress) probably produced an artifactually low TID ratio. The TID ratio was still within the normal pooled range but the heart rate stratified normal range categorized the TID ratio as elevated, consistent with the perfusion pattern of multivessel ischemia. The patient sustained an acute myocardial infarction 11 months after the scan and was found to have severe triple-vessel coronary artery disease at the time of coronary angiography. In two additional cases where the TID ratio was normal according to the conventional pooled upper limit but exceeded a rate-stratified upper limit, coronary angiography performed subsequent to the scan confirmed severe triple-vessel coronary artery disease. One (TID ratio 1.20, change in heart rate 15 BPM) underwent successful percutaneous intervention for a severe proximal LAD stenosis and the other (TID ratio 1.20, change in heart rate 16 BPM) required surgical revascularization.

## Discussion

This study shows that differences in heart rate between the stress and rest sestamibi myocardial perfusion scans are associated with indices of left ventricular volume such as the TID ratio. A direct physiologic link cannot be defined from this study, and further work would be required to establish the mechanism. If venous return remains constant, then changes in heart rate must be accompanied by a reciprocal change in stroke volume. It is not surprising that heart rate effects on left ventricular volume measurements can be identified under conditions of overt arrhythmia. Several cases of falsely elevated and falsely reduced TID ratio have previously been reported [15]. What is surprising in the current study is that this effect is clearly evident even within the range of normal variation in heart rate, though it still only accounted for a minority of the variation in TID ratio. This association does not prove that the variation in heart rate is producing an actual change in ventricular volumes, however. Diastole is disproportionately prolonged at slower heart rates, and end diastolic data would be expected to make a greater contribution to the summed left ventricular data (the basis for the QPS estimation of TID whether acquisitions are gated or non-gated). This could in turn artifactually elevate the summed left ventricular volume at slower heart rates and potentially contribute to the effect of heart rate on the TID ratio. If this were the sole explanation then one might expect that gated SPECT acquisition from our normals (Group 1) would show a negative correlation between heart and summed left ventricular volume but not with end-diastolic volume (EDV) or end-systolic volume (ESV). In fact, the negative correlation existed between all three measures and heart rate was only marginally stronger with the summed volume (summed volume r = -0.27, p = 0.001; EDV r = -0.22, p = 0.007; ESV r = -0.18, p = 0.03). Therefore, the mechanism(s) may be more complicated and needs to be further clarified.

It is not surprising that this effect has not been reported from centres that use a thallium-sestamibi dual-isotope protocol or thallium stress-redistribution imaging [27, 28]. Rest and stress imaging are both completed within a very short period of time and there is probably little opportunity for observing significant temporal or normal changes in heart rate. This is consistent with published reports that the normal range for single-tracer stress/rest sestamibi examinations completed on two days (less than 1.25) is slightly greater than the normal range for a one-day single-tracer rest/stress sestamibi examination (less than 1.18) [29, 30]. These small differences appear to be important in categorizing the TID ratio. We found disagreement in the characterization of the TID ratio as elevated in 8 of 21 cases, indicating possible confounding by heart rate variation. This could have important clinical implications, since one group has reported that an elevated TID ratio is an independent and incremental prognostic marker of cardiac events even in patients with otherwise normal myocardial perfusion scans [31].

A limitation of this study is the lack of clinical outcome data. Whether a heart rate adjusted TID ratio would be superior to the unadjusted TID ratio for the prediction of ischemia-related events is unclear. In theory, a TID ratio whose interpretation is not modified by simple differences in heart rate should provide a more accurate and powerful predictive measure but this still needs to be verified. However, in some patients, heart rate change between stress and rest studies may affect measured left ventricular volumes, and therefore give an erroneous TID ratio, leading to a false positive diagnosis of ischemia, or conversely, a falsely normal value. Our study did not include a true normal population of low-risk individuals. Some of the individuals with normal left ventricular perfusion and function (Group 1) probably had mild coronary artery disease, and this could bias our results. Our use of the peripheral pulse for assessing heart rate on the non-gated scan is obviously prone to error. Electrocardiographic measurement of heart rate for both the stress and rest scans would have been preferable, but this is not routine practice in many nuclear medicine departments. The timing between tracer injection and scanning was not carefully standardized, and could affect the heart rate assessment especially for the stress portion. Therefore, we may have underestimated the effect of heart rate variability on the TID ratio due to inaccuracy in heart rate measurement. For examinations completed on two days there was a greater average change in heart rate (stress heart rate faster than rest heart rate) than when a one day protocol was used. In part, this may reflect the effects of temporarily withdrawing rate-limiting anti-ischemic medication for the stress test. Unfortunately, we do not have information about patient medication use immediately prior to the stress and rest scans.

## Conclusion

In summary, normal variation in heart rate between the stress and rest components of myocardial perfusion scans is common and can influence TID ratios in normal and abnormal cardiac scans. Further work is required to determine if rate specific reference ranges for TID ratio will improve the predictive value of this important index.

## Competing interests

The author(s) declare that they have no competing interests.

## Authors' contributions

WDL conceived the study, performed the data analysis, and drafted the initial article

DPL participated in study design, data interpretation, and revision of the final article

SJD participated in study design, data interpretation, and revision of the final article

All authors read and approved the final manuscript.

## Pre-publication history

The pre-publication history for this paper can be accessed here:



## References

[B1] Berman DS, Hachamovitch R, Kiat H, Cohen I, Cabico JA, Wang FP, Friedman JD, Germano G, Van Train K, Diamond GA (1995). Incremental value of prognostic testing in patients with known or suspected ischemic heart disease: a basis for optimal utilization of exercise technetium-99m sestamibi myocardial perfusion single-photon emission computed tomography. J Am Coll Cardiol.

[B2] Hachamovitch R, Berman DS, Kiat H, Cohen I, Cabico JA, Friedman J, Diamond GA (1996). Exercise myocardial perfusion SPECT in patients without known coronary artery disease: incremental prognostic value and use in risk stratification. Circulation.

[B3] Hachamovitch R, Berman DS, Shaw LJ, Kiat H, Cohen I, Cabico JA, Friedman J, Diamond GA (1998). Incremental prognostic value of myocardial perfusion single photon emission computed tomography for the prediction of cardiac death: differential stratification for risk of cardiac death and myocardial infarction. Circulation.

[B4] Iskander S, Iskandrian AE (1998). Risk assessment using single-photon emission computed tomographic technetium-99m sestamibi imaging. J Am Coll Cardiol.

[B5] Klocke FJ, Baird MG, Lorell BH, Bateman TM, Messer JV, Berman DS, O'Gara PT, Carabello BA, Russell RO, Cerqueira MD, John Sutton MG, DeMaria AN, Udelson JE, Kennedy JW, Verani MS, Williams KA, Antman EM, Smith SC, Alpert JS, Gregoratos G, Anderson JL, Hiratzka LF, Faxon DP, Hunt SA, Fuster V, Jacobs AK, Gibbons RJ, Russell RO (2003). ACC/AHA/ASNC guidelines for the clinical use of cardiac radionuclide imaging--executive summary: a report of the American College of Cardiology/American Heart Association Task Force on Practice Guidelines (ACC/AHA/ASNC Committee to Revise the 1995 Guidelines for the Clinical Use of Cardiac Radionuclide Imaging). J Am Coll Cardiol.

[B6] Burns RJ, Gibbons RJ, Yi Q, Roberts RS, Miller TD, Schaer GL, Anderson JL, Yusuf S (2002). The relationships of left ventricular ejection fraction, end-systolic volume index and infarct size to six-month mortality after hospital discharge following myocardial infarction treated by thrombolysis. J Am Coll Cardiol.

[B7] De Winter O, De Sutter J, Dierckx RA (2002). Clinical relevance of left ventricular volume assessment by gated myocardial SPET in patients with coronary artery disease. Eur J Nucl Med Mol Imaging.

[B8] Sharir T, Germano G, Kang X, Lewin HC, Miranda R, Cohen I, Agafitei RD, Friedman JD, Berman DS (2001). Prediction of myocardial infarction versus cardiac death by gated myocardial perfusion SPECT: risk stratification by the amount of stress-induced ischemia and the poststress ejection fraction. J Nucl Med.

[B9] Hansen CL, Sangrigoli R, Nkadi E, Kramer M (1999). Comparison of pulmonary uptake with transient cavity dilation after exercise thallium-201 perfusion imaging. J Am Coll Cardiol.

[B10] Leslie WD, Tully SA, Yogendran MS, Ward LM, Nour KA, Metge CJ (2005). Prognostic value of lung sestamibi uptake in myocardial perfusion imaging of patients with known or suspected coronary artery disease. J Am Coll Cardiol.

[B11] McLaughlin MG, Danias PG (2002). Transient ischemic dilation: a powerful diagnostic and prognostic finding of stress myocardial perfusion imaging. J Nucl Cardiol.

[B12] Romanens M, Gradel C, Saner H, Pfisterer M (2001). Comparison of 99mTc-sestamibi lung/heart ratio, transient ischaemic dilation and perfusion defect size for the identification of severe and extensive coronary artery disease. Eur J Nucl Med.

[B13] Abidov A, Bax JJ, Hayes SW, Hachamovitch R, Cohen I, Gerlach J, Kang X, Friedman JD, Germano G, Berman DS (2003). Transient ischemic dilation ratio of the left ventricle is a significant predictor of future cardiac events in patients with otherwise normal myocardial perfusion SPECT. J Am Coll Cardiol.

[B14] Kirkpatrick I, Leslie WD (2005). Erroneous left ventricular cavity size measurements on myocardial perfusion SPECT due to transient arrhythmias. Clin Nucl Med.

[B15] Leslie WD, Tully SA, Yogendran MS, Ward LM, Nour KA, Metge CJ (2004). Automated quantification of 99mTc sestamibi myocardial perfusion compared with visual analysis. Nucl Med Commun.

[B16] Germano G, Kavanagh PB, Chen J, Waechter P, Su HT, Kiat H, Berman DS (1995). Operator-less processing of myocardial perfusion SPECT studies. J Nucl Med.

[B17] Germano G, Kavanagh PB, Su HT, Mazzanti M, Kiat H, Hachamovitch R, Van Train KF, Areeda JS, Berman DS (1995). Automatic reorientation of three-dimensional, transaxial myocardial perfusion SPECT images. J Nucl Med.

[B18] Germano G, Kavanagh PB, Berman DS (1997). An automatic approach to the analysis, quantitation and review of perfusion and function from myocardial perfusion SPECT images. Int J Card Imaging.

[B19] Germano G, Kavanagh PB, Waechter P, Areeda J, Van Kriekinge S, Sharir T, Lewin HC, Berman DS (2000). A new algorithm for the quantitation of myocardial perfusion SPECT. I: technical principles and reproducibility. J Nucl Med.

[B20] Sharir T, Germano G, Waechter PB, Kavanagh PB, Areeda JS, Gerlach J, Kang X, Lewin HC, Berman DS (2000). A new algorithm for the quantitation of myocardial perfusion SPECT. II: validation and diagnostic yield. J Nucl Med.

[B21] Leslie WD, Tully SA, Yogendran MS, Ward LM, Nour KA, Metge CJ (2005). Prognostic Value of Automated Quantification of 99mTc-Sestamibi Myocardial Perfusion Imaging. J Nucl Med.

[B22] Sharir T, Germano G, Kavanagh PB, Lai S, Cohen I, Lewin HC, Friedman JD, Zellweger MJ, Berman DS (1999). Incremental prognostic value of post-stress left ventricular ejection fraction and volume by gated myocardial perfusion single photon emission computed tomography. Circulation.

[B23] Duarte PS, Smanio PE, Oliveira CA, Martins LR, Mastrocolla LE, Pereira JC (2003). Clinical significance of transient left ventricular dilation assessed during myocardial Tc-99m sestamibi scintigraphy. Arq Bras Cardiol.

